# Cardiovascular risk factors, ethnicity and infection stone are independent factors associated with reduced renal function in renal stone formers

**DOI:** 10.1371/journal.pone.0265510

**Published:** 2022-04-14

**Authors:** Seow Huey Choy, Selina Ann Nyanatay, Selvalingam Sothilingam, Rohan Malek, Sathiyananthan J. R., Charng Chee Toh, Murali Sundram, Noor Ashani Md Yusoff, Poongkodi Nagappan, Shakirin Kamaruzaman, Wei Sien Yeoh, Teng Aik Ong, Jasmine Lim

**Affiliations:** 1 Department of Surgery, Faculty of Medicine, University of Malaya, Kuala Lumpur, Malaysia; 2 Department of Urology, Hospital Selayang, Selangor, Malaysia; 3 Department of Urology, Hospital Kuala Lumpur, Kuala Lumpur, Malaysia; International University of Health and Welfare, School of Medicine, JAPAN

## Abstract

**Background:**

Recent evidence suggested the link between nephrolithiasis and renal function impairment. We aimed to determine the renal function profile and potential factors associated with reduced renal function amongst renal stone formers in multi-ethnic Asians.

**Methods:**

We conducted a cross-sectional study involving patients undergoing percutaneous nephrolithotomy between May 2015 and December 2019. Reduced renal function was defined as having estimated glomerular filtration rate < 60 ml/min per 1.73 m^2^. Renal stone samples were collected and quantified using infrared spectroscopy. Potential factors associated with reduced renal function including age, ethnicity, educational level, history of diabetes, hypertension, gout, hydronephrosis, serum uric acid level, and type of renal stone were evaluated using univariable and multivariable analyses.

**Results:**

A total of 1162 patients from a multi-ethnic population (Malays 67%, Chinese 19%, Indians 13% and indigenous people 1%) with median age of 57 years (Interquartile range 48–64) were enrolled in the study. Almost a third of patients were found with reduced renal function. Multivariable analysis showed that the odds of having reduced renal function increased with age, ethnicity, lower educational level, history of diabetes, hypertension, gout, bilateral hydronephrosis, elevated serum uric acid level and infection stone.

**Conclusions:**

Reduced renal function varies between ethnicities and all age groups of renal stone formers. In addition to age and ethnicity, cardiovascular risk factors including diabetes and hypertension may also need to be taken into account in managing stone patients with reduced renal function.

## Introduction

Nephrolithiasis is a highly prevalent disease with rising trend observed globally in recent decades [1, 2]. Its prevalence rate varies depending on the study populations across different regions and social constructs [3, 4]. For instance, the burden of nephrolithiasis was 10.6% in the United States [1], 6.2%– 9.1% in Saudi Arabia [5, 6], 6.4% in China [2] and 7.9% in India [7]. Although this disease is more common in males than females in a ratio of 3:1, there is a declining trend of male predominance [8]. Stone recurrence is common. Recurrence of symptomatic renal stones was 11% at 2 years and increased to 39% at 15 years [9]. There is emerging evidence showing a higher incidence of chronic kidney disease (CKD) among stone formers compared to non-stone formers [10, 11].

The global prevalence of CKD was estimated at 9.1% in 2017, resulting in 1.2 million deaths [12]. It is a long-term illness which may progress from mild reduction in glomerular filtration rate to end-stage renal disease (ESRD) without appropriate treatment. In Malaysia, the CKD prevalence was 15.5% in 2018; of which, 6.8% had estimated glomerular filtration rate (eGFR) < 60 ml/min/1.73m^2^ [13]. The expenditure of Malaysia healthcare system recorded an average annual growth of 12% with average USD 575 million per year in ESRD between 2010 and 2016 [14]. Based on the 2012 Kidney Disease Improving Global Outcomes (KDIGO) guidelines, individuals with eGFR <60 ml/min/1.73m^2^ have moderate to very high risks of developing CKD compared to those of eGFR ≥60 ml/min/1.73m^2^, [15]. Whilst full evaluation of CKD progression relies on KDIGO definition, assessment of GFR and albuminuria level, an abnormal eGFR could be an early indicator of CKD.

Recent emerging evidence identified nephrolithiasis as one of the risk factors for renal function impairment; however, it remains uncertain whether this risk differs by stone type. For example, lower urinary pH was usually present in individuals with metabolic syndrome, hyperuricemia and gout, predisposing them to the formation of uric acid stones [16, 17]. Defective urinary ammonium excretion, which contributes to the persistent acidic urine, could lead to impaired renal function in uric acid stone formers [18]. It has been hypothesised that rare monogenetic disorders and malformations such as primary hyperoxaluria and cystinuria can cause stone formation and increase the risk for renal function loss [10]. Previous study demonstrated the association of high urinary oxalate excretion with CKD progression [19]. Formation of calcium oxalate crystal, obstruction and damage to the tubular epithelial cells as well as inflammation of tissue parenchyma under high oxalate concentration can result in kidney injury [19]. Therefore, it is of our interest to assess the renal function profile among renal stone patients and identify potential factors associated with reduced renal function in a multi-ethnic Asian setting.

## Materials and methods

### Study participants

This cross-sectional study was conducted in three tertiary urology referral centres including Hospital Kuala Lumpur, Hospital Selayang and University Malaya Medical Centre, Malaysia. Patients undergoing percutaneous nephrolithotomy (PCNL) for renal calculi between May 2015 and December 2019 were enrolled into the study. Of note, these patients usually present with relatively large stone (>1.5cm) and are at higher risk of developing complications including renal failure and sepsis. We excluded patients with Karnofsky Performance Score ≤70 from the study. All participants provided written informed consent. The study protocol was approved by the Medical Research and Ethics Committee at UMMC (code: MECID. no 20152–1020) and Ministry of Health Malaysia (code: NMRR-15-35-24341).

### Data collection and measurements

A structured questionnaire consisting of sociodemographic and clinical parameters was used. Basic demographic included age (year), gender (male and female), ethnicity (Malay, Chinese, and Indian), marital status (married and single), educational level, smoking status, and alcohol consumption. Educational level was grouped into tertiary, secondary or primary, and nonformal. Smoking status was categorised into no (never smoked or ex-smoker who had discontinued >1 month) and yes (smoking on daily or occasional basis). Alcohol consumption was divided into no (never or less than once a month) and yes (more than once a month).

Clinical information included weight (kg), height (cm), waist circumference (cm), family history of renal stone, history of major comorbidities such as diabetes, dyslipidaemia hypertension, gout, hydronephrosis, serum creatinine (μmol/L) level, serum uric acid level (elevated uric acid level was defined as uric acid >430 μmol/L for male and uric acid >360 mol/L for female) [20], stone episode (new and recurrence), stone location (non-staghorn and staghorn or partial staghorn), and number of stones (single and multiple). Central obesity was defined as having waist circumference >79.9 cm for female and waist circumference >89.9 cm for male [21]. Renal function was evaluated using eGFR based on the CKD Epidemiology Collaboration equation (CKD-EPI) [22]. The CKD-EPI equation was previously validated in a multi-ethnic population in Singapore [23]. Reduced renal function was defined as eGFR <60 ml/min per 1.73 m^2^ based on the KDIGO guidelines [15].

Overnight fasting blood sample was obtained to measure lipid profile and fasting blood glucose level using Advin 2400 (Siemens Healthcare Diagnostic Inc, Muenchen, Germany). The HbA1c level was analysed with VARIANTTM II Hemoglobin Testing System (Bio-Rad Laboratories, Inc, California, United States). Patients with HDL <1.03 mmol/L (male); HDL <1.29 mmol/L (female) or triglyceride ≥1.69 mmol/L or self-reported use of lipid-lowering drugs were defined as having dyslipidaemia [24]. Patients displaying fasting blood glucose levels ≥7.0 mmol/L or HbA1c ≥6.3% (≥45 mmol/mol) or self- reported on treatment for diabetes were considered to have diabetes mellitus [25].

Renal stone samples were collected after the PCNL procedure and analysed quantitatively in a central laboratory using Nicolet iS5 Fourier-transform infrared (FT-IR) spectrometer (Thermo Fisher Scientific Inc, Waltham, USA) to investigate the stone composition. Stone type was classified into calcium oxalate, infection, uric acid, and cystine based on the predominant stone composition, accounting for >55% of the overall stone composition. Infection stones were magnesium ammonium phosphate hexahydrate (struvite), ammonium urate, and carbonate apatite. A mixed stone was a renal stone which did not consist of a major component accounting for more than 55%.

### Statistical analysis

Continuous variables were expressed as median and interquartile range (IQR) whilst categorical variables were expressed in frequencies and percentages. Potential factors associated with reduced renal function were compared between two groups, alone and in combination, using univariable and multivariable logistic regression. The missing data in most variables were between 1.5–4.1% except serum uric acid level (18.1%), central obesity (10.2%) and renal stone type (15.2%). We assumed the missingness to occur completely at random. All statistical analyses were conducted using Statistic Package for Social Science (SPSS) software for Windows Version 27 (SPSS Inc, Chicago, Illinois, USA). Two-tailed *p* value <0.05 was termed statistically significant.

## Results

A total of 1162 renal stone patients undergoing PCNL procedure were included into the study, consisting of 55% males and 45% females. Most of the patients were Malays (n = 778; 67.0%), followed by Chinese (n = 222; 19.1%), Indians (n = 152; 13.1%), and indigenous people (n = 10; 0.9%) with median age of 57 years (IQR 48–64). The commonest stone type was calcium oxalate (n = 526; 53.4%), followed by infection stone (n = 204; 20.7%) and uric acid stone (n = 165; 16.8%). Majority patients were first-time stone formers (69.9%) and approximately half of the stone formers developed staghorn or partial staghorn stones. In term of stone type, patients with calcium oxalate stones had the highest median eGFR (81.30 ml/min/1.73m^2^, IQR 57.70–95.89) whilst patients with cystine stone had the lowest median eGFR (61.12 ml/min/1.73m^2^, IQR 50.55–71.55). The overall median eGFR was 77.15 ml/min/1.73m^2^ (IQR 52.28–94.75) with higher median eGFR observed among younger patients aged ≤49 compared to those >49 (Table 1). The median eGFR of Malays (72.38 ml/min/1.73m^2^, IQR 48.29–93.54) was relatively lower than Chinese (81.35 ml/min/1.73m^2^, IQR 60.71–93.36), Indians (88.42ml/min/1.73m^2^, IQR 66.38–101.61), and indigenous people (88.95 ml/min/1.73m^2^, IQR 69.60–105.35).

**Table 1:** The estimated glomerular filtration rate of stone formers.

Variable	n (%)	eGFR (ml/min/1.73m^2^)	
		Median	Interquartile range
**Age**			
≤49	320 (27.5)	93.10	75.68–108.85
50–59	364 (31.3)	76.90	55.15–95.18
60–69	378 (32.5)	66.16	45.33–88.47
≥70	100 (8.6)	53.52	37.29–78.30
**Gender**			
Male	639 (55.0)	75.40	52.50–93.33
Female	523 (45.0)	79.70	51.90–97.20
**Ethinicity**			
Malay	778 (67.0)	72.38	48.29–93.54
Chinese	222 (19.1)	81.35	60.71–93.36
Indian	152 (13.1)	88.42	66.38–101.61
Indigenous	10 (0.9)	88.95	69.60–105.35
**Stone type**			
Calcium oxalate	526 (53.4)	81.30	57.70–95.89
Infection	204 (20.7)	76.45	46.23–96.10
Uric acid	165 (16.8)	67.17	43.45–87.05
Cystine	6 (0.6)	61.12	50.55–71.55
Others ^a^	84 (8.5)	75.00	46.63–99.80

^a^ Others consisted of mixed, calcium phosphate, and rare stones

Abbreviations: eGFR, estimated glomerular filtration rate.

Table 2 summarises the characteristics of patients categorised into normal renal function (eGFR ≥60 ml/min/1.73m^2^) and reduced renal function (eGFR <60 ml/min/1.73m^2^). Overall, there were 31.7% (368/1162) of stone formers with reduced renal function. We found significant association between reduced renal function and age, ethnicity, marital status, educational level, smoking status, history of diabetes, dyslipidaemia, hypertension, gout, and bilateral hydronephrosis as well as serum uric acid level and stone type (*p* <0.05) (Table 2). For instance, patients with history of hypertension or diabetes were 2.9 fold and 1.83 fold more likely to have reduced renal function respectively, compared to those without these medical conditions. It was revealed that patients with gout were 3 times more likely to have reduced renal function compared with patients without gout. Patients with elevated serum uric acid level were 3.59 times more likely than those with normal serum uric acid level to have reduced renal function (*p*<0.001).

**Table 2:** Comparison of factors associated with renal function defined by eGFR.

	Frequency distribution, n (%)					
Variable	Overall	Normal renal function (eGFR ≥60 ml/min/1.73m^2^)	Reduced renal function (eGFR <60 ml/min/1.73m^2^)	OR	95% CI	P value
***Sociodemographic characteristics***						
**Age**, year	1162 (100)	794 (68.3)	368 (31.7)	1.06	1.05–1.07	**<0.001**
Median (IQR)	57 (48–64)	54 (46–62)	62 (55–67)			
**Gender**						
Male	639 (55.0)	442 (55.7)	197 (53.5)	1.00		
Female	523 (45.0)	352 (44.3)	171 (46.5)	1.09	0.85–1.40	0.496
**Ethnicity**						
Malay	778 (67.0)	499 (62.8)	279 (75.8)	1.00		
Chinese	222 (19.1)	168 (21.2)	54 (14.7)	0.58	0.41–0.81	**0.001**
Indian	152 (13.1)	119 (15.0)	33 (9.0)	0.50	0.33–0.75	**0.001**
Indigenous	10 (0.9)	8 (1.0)	2 (0.5)	0.45	0.09–2.12	0.311
**Marital status**						
Married	1033 (91.1)	694 (89.7)	339 (94.2)	1.00		
Single	101 (8.9)	80 (10.3)	21 (5.8)	0.54	0.33–0.88	**0.014**
*Unknown*	28	20	8			
**Educational level**						
Tertiary	131 (11.5)	107 (13.7)	24 (6.6)	1.00		
Primary or secondary	945 (82.6)	645 (82.5)	300 (82.9)	2.07	1.31–3.30	**0.002**
None	68 (5.9)	30 (3.8)	38 (10.5)	5.65	2.94–10.84	**<0.001**
*Unknown*	18	12	6			
**Smoking status**						
No	909 (79.9)	605 (77.7)	304 (84.7)	1.00		
Yes	229 (20.1)	174 (22.3)	55 (15.3)	0.63	0.45–0.88	**0.006**
U*nknown*	24	15	9			
**Alcohol consumption**						
No	976 (85.4)	665 (85.5)	311 (85.2)	1.00		
Yes	167 (14.6)	113 (14.5)	54 (14.8)	1.02	0.72–1.45	0.904
*Unknown*	19	16	3			
***Clinical characteristics***						
**Family history of renal stone**						
No	771 (69.1)	526 (68.9)	245 (69.4)	1.00		
Yes	345 (30.9)	237 (31.1)	108 (30.6)	0.98	0.74–1.29	0.875
*Unknown*	46	31	15			
**History of diabetes^a^**						
No	553 (47.6)	415 (52.3)	138 (37.5)	1.00		
Yes	609 (52.4)	379 (47.7)	230 (62.5)	1.83	1.42–2.35	**<0.001**
**History of dyslipidaemia^b^**						
No	333 (28.7)	242 (30.5)	91 (24.7)	1.00		
Yes	829 (71.3)	552 (69.5)	277 (75.3)	1.33	1.01–1.77	**0.044**
**History of hypertension**						
No	351 (30.2)	290 (36.5)	61 (16.6)	1.00		
Yes	811 (69.8)	504 (63.5)	307 (83.4)	2.90	2.12–3.95	**<0.001**
**History of gout**						
No	1095 (94.2)	765 (96.3)	330 (89.7)	1.00		
Yes	67 (5.8)	29 (3.7)	38 (10.3)	3.04	1.84–5.01	**<0.001**
**Serum uric acid**						
Normal	532 (55.9)	422 (66.0)	110 (35.1)	1.00		
Elevated ^c^	420 (44.1)	217 (34.0)	203 (64.9)	3.59	2.70–4.77	**<0.001**
*Unknown*	210	155	55			
**History of hydronephrosis**						
No	416 (36.4)	289 (37.0)	127 (35.0)	1.00		
Unilateral	620 (54.2)	436 (55.8)	184 (50.7)	0.96	0.73–1.26	0.769
Bilateral	108 (9.4)	56 (7.2)	52 (14.3)	2.11	1.37–3.25	**0.001**
*Unknown*	18	13	5			
***Anthropometric measurements***						
**Central obesity Asian ^d^**						
No	254 (24.4)	179 (24.8)	75 (23.4)	1.00		
Yes	789 (75.6)	543 (75.2)	246 (76.6)	1.08	0.79–1.47	0.620
*Unknown*	119	72	47			
**Body mass index, kg/m^2^, Median (IQR)**	27.1 (24.2–30.5)	27.1 (24.0–30.4)	27.1 (24.4–31.0)	1.00	0.99–1.01	0.576
***Stone-specific parameters***						
**Stone episode**						
New	797 (69.9)	550 (70.2)	247 (69.0)	1.00		
Recurrence	344 (30.1)	233 (29.8)	111 (31.0)	1.06	0.81–1.39	0.670
*Unknown*	21	11	10			
**Stone location**						
Non-staghorn	586 (51.1)	409 (52.0)	177 (49.2)	1.00		
Staghorn/Partial staghorn	560 (48.9)	377 (48.0)	183 (50.8)	1.12	0.87–1.44	0.367
*Unknown*	16	8	8			
**Number of stones**						
Single	480 (43.1)	336 (44.2)	144 (40.7)	1.00		
Multiple	634 (56.9)	424 (55.8)	210 (59.3)	1.16	0.90–1.49	0.268
*Unknown*	48	34	14			
**Renal stone type**						
Calcium oxalate	526 (53.4)	382 (57.3)	144 (45.3)	1.00		
Infection	204 (20.7)	132 (19.8)	72 (22.6)	1.45	1.03–2.04	**0.036**
Uric acid	165 (16.8)	98 (14.7)	67 (21.1)	1.81	1.26–2.61	**0.001**
Others ^e^	90 (9.1)	55 (8.2)	35 (11.0)	1.69	1.06–2.69	**0.027**
*Unknown*	177	127	50			

^a^FBG ≥7.0 mmol/L or HbA_1c_ ≥6.3% (≥45 mmol/mol) or self-reported diabetic on treatment [25]

^b^HDL<1.03 mmol/L (male), <1.29 mmol/L (female) or Triglyceride ≥1.69 mmol/L or self-reported on treatment [24]

^c^Uric acid >430 μmol/L (male); >360 mol/L (female)[20]

^d^Waist circumference >79.9 cm (female); >89.9 cm (male) [21]

^e^Mixed stone, calcium phosphate, cystine, and rare stone

Abbreviations: CI, confidence interval; eGFR, estimated glomerular filtration rate; FBG, fasting blood glucose; HDL, high-density lipoprotein; IQR, interquartile range; OR, odds ratio; SD, standard deviation

In the multivariable analysis, age, ethnicity, education level, history of diabetes, hypertension, gout, and bilateral hydronephrosis as well as serum uric acid level and renal stone type remained to be significantly and independently associated with reduced renal function amongst stone formers (Table 3). Patients with infection stone [adjusted odds ratio (aOR) 1.83, 95% confidence interval (CI) 1.22–2.74] were more likely to have reduced renal function than those with calcium oxalate stone. Comparing to Malays, Chinese and Indians had 58% and 41% lesser odds of having reduced renal function respectively. Increased odds of having reduced renal function was observed in stone patients with history of bilateral hydronephrosis (aOR 3.23, 95% CI 1.95–5.34).

**Table 3:** Multivariable analysis of factors associated with reduced renal function (eGFR <60 ml/min/1.73m^2^).

Variable	Regression coefficient	aOR	95% CI	P value
**Age**	0.064	1.07	1.05–1.08	**<0.001**
**Ethnicity**				
Malay	Reference			
Chinese	-0.867	0.42	0.28–0.63	**<0.001**
Indian	-0.530	0.59	0.37–0.94	**0.028**
Indigenous	-0.088	0.92	0.16–5.21	0.921
**Marital status**				
Married	Reference			
Single	0.511	1.67	0.93–2.99	0.085
**Educational level**				
Tertiary	Reference			
Primary or secondary	0.546	1.73	1.02–2.92	**0.042**
None	1.262	3.53	1.65–7.56	**0.001**
**Smoking status**				
No	Reference			
Yes	-0.035	0.97	0.65–1.43	0.859
**History of diabetes**				
No	Reference			
Yes	0.313	1.37	1.01–1.87	**0.048**
**History of dyslipidaemia**				
No	Reference			
Yes	0.256	1.29	0.93–1.80	0.131
**History of hypertension**				
No	Reference			
Yes	0.494	1.64	1.12–2.39	**0.010**
**History of gout**				
No	Reference			
Yes	0.891	2.44	1.37–4.34	**0.002**
**History of hydronephrosis**				
No	Reference			
Unilateral	0.186	1.21	0.88–1.65	0.245
Bilateral	1.172	3.23	1.95–5.34	**<0.001**
**Serum uric acid**				
Normal	Reference			
Elevated	1.209	3.35	2.43–4.61	**<0.001**
**Renal stone type**				
Calcium oxalate	Reference			
Infection	0.604	1.83	1.22–2.74	**0.003**
Uric acid	0.250	1.28	0.83–1.99	0.261
Others	0.878	2.41	1.38–4.20	**0.002**

Abbreviations: CI, confidence interval; eGFR, estimated glomerular filtration rate; aOR, adjusted odds ratio.

## Discussion

Findings from this study provide new insights into the severity of reduced renal function in multi-ethnic renal stone formers. We demonstrated ethnic variations, major stone elements and cardiovascular risk factors especially diabetes and hypertension were significantly associated with reduced renal function in renal stone patients. The relative high prevalence of reduced renal function was in line with previous study recording 39.2% of stone formers having eGFR <60 ml/min/1.73m^2^ [26]. Although nephrolithiasis is usually presented as a benign medical condition with limited long-term consequences, high prevalence of reduced renal function found in our study is alarming as previous meta-analyses demonstrated significant associations of renal stones with higher risk of CKD [27, 28]. An increased risk of CKD with an adjusted risk ratio of 1.47 (95% CI 1.23–1.76) was estimated in patients with renal stone history [27]. Our findings revealed that sociodemographic factors such as ethnicity and lower education level were associated with reduced renal function among stone formers. These are in parallel with previous studies showing association of CKD ethnicity and socioeconomic status [29], owing to dietary patterns and health-related behaviours.

In the present study, high prevalence of cardiovascular risk factors such as diabetes, dyslipidaemia, hypertension, and central obesity were found among stone formers (ranging 52.2% to 75.4%). These cardiovascular risk factors except central obesity were associated with reduced renal function in the univariable analysis. Diabetes and hypertension remained as significant factors after multivariable adjustment. There are emerging evidence showing association of cardiovascular risk factors and its individual components with CKD and nephrolithiasis, alone [30–32] and in combination [33, 34]. The prevalence of CKD within Malaysian population increased from 9.1% in 2011 to 15.5% in 2018, attributing to the rising prevalence of non-communicable diseases and aging population in Malaysia [13]. The National Health and Morbidity Survey (NHMS) showed diabetic patients increased from 11.6% in 2006 to 18.3% in 2019 while the prevalence of raised blood cholesterol was 38.1% in 2019 compared to 20.7% in 2006 [35, 36]. Stone formers with reduced renal function in the present study were older than those of normal renal function (60 years old vs. 52 years old), suggesting old age may be responsible in increasing the risk of comorbidities related to CKD or increasing the susceptibility of stone formers with multiple comorbid conditions to CKD [33].

Hyperuricemia and gout were associated with nephrolithiasis [37, 38]. We observed a statistically significant association of reduced renal function with gout and increased serum uric acid level. This is consistent with previous study showing higher prevalence of hyperuricemia (aOR 9.8, 95% CI 4.3–22.0) and gout (aOR 5.9, 95% CI 2.2–15.7) among individuals with severe renal impairment compared to those without renal impairment in the US general population [39]. Uric acid is the by-product of exogenous and endogenous purine metabolism that usually exists in the form of salt as urate. Kidneys excrete about two-thirds of the uric acid load [40]. The rise of urid acid production and impaired renal uric acid excretion could result in hyperuricemia [41]. Hyperuricemia is therefore generally considered as a complication of renal dysfunction [40]. Interestingly, recent studies suggested the pathogenic role of uric acid in CKD progression rather than being a marker of impaired renal uric acid excretion alone [42]. In a cohort study involving 10,677 individuals with renal function eGFR ≥60 ml/min/1.73m^2^ and negative proteinuria, patients with increased serum uric acid level had greater odds of developing renal disease which defined as an incidence of a decline in eGFR from ≥ 30% at baseline to <60 ml/min/1.73m^2^ [43]. It was also demonstrated that the use of allopurinol or other urate-lowering therapy was able to slow the progression of renal disease in patients with hyperuricemia [44], CKD [45, 46] and gout [47].

In this study, we showed that infection stone were associated with reduced renal function, which is consistent with findings from previous studies [33, 48, 49]. It is suggested that loss of renal function amongst infection stone formers was attributed to recurrent urinary infection and high growth rate, occupying the entire renal collecting system rapidly [50, 51]. The level of renal function varies across different stones types. Uric acid stone formers had the lowest median eGFR after cystine stone formers in our study. This finding was in line with a previous study showing uric acid stone formers had significantly worse renal function compared to calcium stone formers or individuals without renal stone regardless of hyperuricemia status [18]. Chou *et al*. reported that renal function was significantly better in patients with calcium stones (i.e. calcium oxalate and calcium phosphate) than those of struvite or uric acid stone [51], suggesting an increased CKD risk amongst non-calcium stone formers. It is possible that urological procedures for stone such as PCNL may contribute to renal damage, prior to CKD stage as well as frequency and complexity of stone treatments [52]. Recurrent or multiple stone events might expose stone formers to higher risk of renal impairment, potentially due to cumulative kidney injury from obstructive uropathy [53].

There are limitations to this multicentre study. First, causal relationships between nephrolithiasis and renal function could not be established in this study due to its cross-sectional study design. Second, the generalisability and extrapolation of these findings to other populations remain unknown. Third, renal function was estimated using eGFR based on a single serum creatinine measurement. This can potentially overestimate the prevalence of reduced renal function as eGFR could be fluctuated in the presence of stone [54]. The level of eGFR pre- and post-PNCL could be considered in the future study. Fourth, addition of more clinical information such as serum level of calcium, vitamin D& parathyroid hormone, urine culture results and history of drug consumption may increase the accuracy of the multivariable analysis.

The strength of this study are the large sample size (>1000 patients) and adequate representation from three major Asian ethnicities. There is a low rate of missing data (1.5–4.1%) except serum uric acid (18.1%), central obesity (10.2%), and renal stone type (15.2%). In addition, all the blood tests including fasting blood glucose, HbA1c and lipid profile as well as renal stone quantitative analysis were conducted in central laboratories to avoid testing discrepancies.

In summary, our study findings concluded that renal stone is a systemic disease with significant association observed between reduced renal function and cardiovascular risk factors such as hypertension and diabetes in a multi-ethnic Asian population. We recommend stone patients to undergo more aggressive screening for subclinical CKD. Similarly, clinicians may consider screening stone patients present with reduced renal function for cardiovascular risk factors.
